# Marginal adaptation and fracture resistance of CAD/CAM-fabricated PMMA, PEEK, and PEKK three-unit provisional fixed dental prostheses: an in vitro comparative study

**DOI:** 10.2340/biid.v13.46737

**Published:** 2026-08-26

**Authors:** Mustafa Mohammad Harfi, Shaimaa Ahmed Abo El-Farag, Ahmed Shams

**Affiliations:** a Fixed Prosthodontics Department, Faculty of Dentistry, Mansoura University, Mansoura, Egypt; b Fixed Prosthodontics Department, Faculty of Dentistry, Mansoura National University, Mansoura, Egypt

**Keywords:** Marginal adaptation, fracture resistance, CAD/CAM, provisional, fixed dental prostheses, PMMA, PEEK, PEKK

## Abstract

**Background:**

Although polymethyl methacrylate (PMMA) is widely used for computer-aided design and computer-aided manufacturing (CAD/CAM) provisional restorations, high-performance polymers such as polyetheretherketone (PEEK) and polyetherketoneketone (PEKK) have been proposed as alternatives. However, limited data are available comparing these materials in multi-unit provisional fixed dental prostheses (FDPs). This in vitro study aimed to evaluate and compare the marginal adaptation and fracture resistance of CAD/CAM-fabricated three-unit provisional FDPs constructed from PMMA, PEEK, and PEKK.

**Materials and Methods:**

A total of 48 sound human maxillary teeth (24 second premolars and 24 second molars) were prepared to receive 24 CAD/CAM-fabricated three-unit provisional FDPs. Specimens were randomly assigned into three groups (*n* = 8) according to material: PMMA, PEEK, and PEKK. Marginal adaptation was initially measured before cementation using a stereomicroscope. Following cementation, all specimens underwent standardized thermomechanical aging (10,000 thermal cycles and 240,000 mechanical loading cycles), after which marginal adaptation was reevaluated. Fracture resistance was then assessed using a universal testing machine, followed by analysis with scanning electron microscopy. Data were statistically analyzed using two-way repeated-measures analysis of variance (ANOVA), one-way ANOVA, Tukey’s post hoc test, and Fisher’s exact test (*P* ≤ 0.05).

**Results:**

Following cementation and aging, PMMA showed significantly lower marginal discrepancy than PEEK and PEKK (*P* < 0.001). PEEK exhibited a significant improvement in marginal adaptation after thermomechanical aging (*P* = 0.002), whereas PMMA and PEKK showed no significant changes. Fracture resistance differed significantly among groups (*P* < 0.001), with PEKK recording the highest mean fracture load (2,669 ± 204 N), followed by PEEK (1,849 ± 183 N), while PMMA showed the lowest mean value (1,072 ± 127 N).

**Conclusions:**

Under the tested experimental conditions, material type significantly affected both marginal adaptation and fracture resistance. Among the tested materials, PMMA demonstrated the lowest marginal discrepancy, and PEKK exhibited the highest fracture resistance, whereas PEEK provided a balanced performance.

KEY MESSAGES

- Material selection matters: The type of CAD/CAM material significantly affects both the marginal adaptation and fracture resistance of three-unit provisional fixed dental prostheses.
- All materials demonstrate favorable performance: All tested PMMA, PEEK, and PEKK materials demonstrated marginal adaptation within the clinically acceptable range and fracture resistance values exceeding the normal posterior masticatory forces.
- Each material offers a distinct performance profile: PMMA demonstrated the lowest marginal discrepancy, PEKK exhibited the highest fracture resistance, whereas PEEK provided a balanced performance in terms of marginal adaptation and fracture resistance.

## Introduction

Provisional fixed dental prostheses (FDPs) are an integral part of prosthodontic treatment, as they protect prepared abutments, maintain occlusal relationships, preserve periodontal health, and provide functional and esthetic stability during the interim treatment phase [1]. The clinical success of provisional restorations depends largely on their marginal adaptation and mechanical strength. Poor marginal adaptation may lead to microleakage, plaque accumulation, and periodontal inflammation, whereas insufficient fracture resistance may result in restoration failure under masticatory forces, particularly in patients with high occlusal loads or parafunctional habits [2].

Polymethylmethacrylate (PMMA) has been widely used for the fabrication of provisional restorations due to its acceptable esthetics, ease of processing, and low cost. However, conventionally fabricated PMMA provisionals are associated with several drawbacks, including polymerization shrinkage, marginal discrepancies, and relatively low fracture resistance, particularly in multi-unit FDPs. These limitations may compromise the fit and longevity of provisional restorations, negatively impacting both patient comfort and periodontal health [3, 4].

The introduction of computer-aided design and computer-aided manufacturing (CAD/CAM) technologies has revolutionized the fabrication of provisional restorations. CAD/CAM allows the use of industrially polymerized blocks with improved mechanical properties, superior dimensional stability, and reduced polymerization shrinkage compared to conventional techniques. As a result, CAD/CAM-fabricated provisionals generally demonstrate improved marginal adaptation, higher fracture resistance, and better overall clinical performance, making them particularly suitable for multi-unit FDPs and complex rehabilitations [5, 6].

High-performance polymers, such as polyetheretherketone (PEEK) and polyetherketoneketone (PEKK), are increasingly being explored in prosthodontics due to their favorable biomechanical characteristics. These materials exhibit high fracture resistance, a low elastic modulus closer to that of bone, excellent fatigue behavior, and chemical stability, which can reduce stress on abutments and improve long-term performance. Additionally, their biocompatibility and low plaque affinity make them promising candidates for both provisional and definitive restorations [7–9]. Accordingly, PMMA was selected in the present study as the conventional CAD/CAM provisional material because it remains the most widely used clinical reference, whereas PEEK and PEKK were selected as representative high-performance polymers due to their promising mechanical and biological properties and their increasing application in digital prosthodontics. Comparing these materials may provide clinically relevant evidence regarding the advantages and limitations of conventional and high-performance CAD/CAM polymers for multi-unit provisional FDPs.

Despite the growing interest in PEEK and PEKK, there is limited evidence directly comparing their performance with conventional PMMA, particularly in CAD/CAM-fabricated multi-unit provisional FDPs. Moreover, previous studies have mainly focused on individual material properties rather than providing a comprehensive comparison of both marginal adaptation and fracture resistance. These two outcomes are critical key factors that collectively influence the clinical performance of provisional FDPs, as optimal restorations should provide adequate marginal sealing for maintaining biological integrity, while simultaneously withstanding functional occlusal loading throughout the provisionalization period. Therefore, a better understanding of how these materials affect both outcomes is clinically important, as it may assist clinicians in selecting the most appropriate provisional restorative material according to specific functional and biological requirements [3, 4]. Accordingly, this in vitro study aimed to evaluate and compare the marginal adaptation and fracture resistance of CAD/CAM-fabricated three-unit provisional FDPs fabricated from PMMA, PEEK, and PEKK. The tested null hypothesis was that there would be no significant differences in marginal adaptation and fracture resistance among provisional FDPs fabricated using these CAD/CAM materials.

## Materials and methods

### Ethical approval

This in vitro study was carried out following the regulations and ethical guidelines established by the Local Research Ethics Committee, Faculty of Dentistry, Mansoura University. Ethical clearance for the study was granted under approval number A0303024FP.

### Sample size calculation

The required sample size was calculated using G*Power^TM^ software (version 3.1.9.7, Heinrich Heine University Düsseldorf, Düsseldorf, Germany). The calculation was based on the marginal adaptation data reported in a previously published study evaluating different provisional materials [10]. A two-tailed power analysis was performed using an effect size of 0.56, a significance level (α) of 0.05, and a statistical power of 80%. The results indicated that a minimum of eight specimens per group was required.

### Tooth selection

A total of 48 sound human teeth, comprising 24 maxillary second premolars and 24 maxillary second molars, were selected for the study. All teeth had fully developed roots and were freshly extracted for periodontal or orthodontic reasons. Teeth with similar dimensions and morphology were chosen to minimize variability. All teeth were obtained with patient consent from the Oral and Maxillofacial Surgery Department, Faculty of Dentistry, Mansoura University. At the cemento-enamel junction (CEJ) level, occlusocervical, buccolingual, and mesiodistal dimensions, as well as root lengths, were measured using a digital caliper (100 mm/4 in Digital Gauge Vernier Caliper^TM^, Hanhe^®^ Co., Ltd., Zhuzhou, China). Teeth exhibiting cracks, caries, restorations, excessive root curvature, or atypical root morphology were excluded. Additionally, all specimens were examined under blue-light transillumination using a magnified dental loupe (KKA088633, Univet^®^ SRL, Brescia, Italy) to ensure the absence of microcracks [11, 12].

### Tooth cleaning, disinfection, and storage

The selected teeth were initially cleaned to remove superficial staining, calculus, and adherent soft tissues using an ultrasonic scaler, followed by low-speed polishing with polishing paste. In accordance with 1993 CDC (Centers for Disease Control and Prevention, Atlanta, Georgia, USA) recommendations, all selected teeth were disinfected by immersion for 1 week in a 1:10 diluted solution of 5.25% sodium hypochlorite household bleach (Clorox^®^ Bleach, Clorox Co., Cairo, Egypt). To prevent dehydration, teeth were stored in 0.9% sterile saline solution at room temperature throughout the experimental period [11, 13].

### Tooth mounting

For standardized handling, the roots of teeth were vertically embedded along their long axes within acrylic resin blocks. As abutments of each studied three-unit FDP, one maxillary second premolar tooth and one maxillary second molar tooth of the selected teeth were mounted separately in a plastic ring filled with pink self-curing acrylic resin material (Acrostone^®^ cold cure denture base material, Acrostone Co., Ltd, Cairo, Egypt) using a 1-arm dental laboratory parallelometer device (Delineador B2^TM^, Bio-Art^®^ Co., SP, Brazil). This surveyor device was used to allow accurate centralization and alignment of both abutment teeth in the ring during block fabrication [14].

For all cleaned selected teeth, two reference lines were marked using a waterproof marker. The first line, representing the simulated alveolar bone level, was drawn 1 mm apical to the CEJ level [14]. The second line was placed 2 mm below the CEJ or 1 mm below the first line and parallel to it, representing the coronal limit of the periodontal ligament (PDL) [15]. PDL simulation was achieved using the ‘’Transitional Wax Technique’’, creating a uniform 0.3 mm space around the roots. A light-body vinyl polysiloxane (VPS) impression material (Perfit^®^ Light-body, Huge Dent, Shandong, China) was used to simulate the PDL layer [11, 14, 16, 17]. A standardized edentulous span of 11 mm was maintained between the premolar and molar abutments to represent the missing maxillary first molar (pontic site) for all FDPs [18]. After complete setting of resin blocks, all specimens were cleaned, polished, and stored in the saline solution at room temperature until the preparation stage. All procedures were performed by a single operator to ensure standardization.

### Specimen grouping

The resulting 24 acrylic resin blocks representing 24 three-unit provisional FDPs were numbered from 1 ascending to 24. They were randomly assigned using RANDOM.ORG^®^ (Randomness and Integrity Services Ltd., Dublin, Ireland) into three equal groups (*n* = 8) according to the CAD/CAM material used for fabrication of provisional FDPs: Group (PMMA): 8 provisional FDPs fabricated using polymethylmethacrylate material (HUGE^®^ PMMA Block), Group (PEEK): 8 provisional FDPs fabricated using polyetheretherketone material (PEEK Dental Disc^TM^), and Group (PEKK): 8 provisional FDPs fabricated using polyetherketoneketone material (PEKKTON^®^ Ivory milling blank). The materials used for the fabrication and cementation of provisional FDPs in this study are listed in Table 1.

**Table 1:** Materials used for the fabrication and cementation of provisional FDPs.

Category	Material	Product name	Batch number	Composition	Manufacturer
The FDP material	PMMA material	HUGE^®^ PMMA Block (98 × 20 mm)	240621010	Polymethylmethacrylate (> 95%), Residual methylmethacrylate (< 1%), Additives (UV stabilizers, pigments) (< 5%)	HUGE Dental Material Co., Ltd., Shandong, China
	PEEK material	PEEK Dental Disc^TM^ (98 × 16 mm)	034520493	Polyetheretherketone (> 95%), Additives (stabilizers, pigments) (< 5%)	Changzhou International Trade Co., Ltd., Changzhou, China
	PEKK material	PEKKTON^®^ Ivory milling blank (98.5/t20 mm)	2023-07-15-CM	- Polyetherketoneketone (PEKK) (90%) - Titanium dioxide (TiO_2_) (10%)	Cendres + Metaux SA, Biel/Bienne, Switzerland.
The luting cement	Zinc oxide eugenol-free temporary cement	Cavex^®^ Temporary Cement	250042	- White Base paste: Metallic oxides (80%), Vegetable oil (20%) - Yellow Catalyst paste: Fatty acid dimer (69%), Natural and modified resins (21%), Organic acid (6%), Natural waxes (3%), Pigment (< 1%)	Cavex Holland BV, Haarlem, Netherlands

FDP: fixed dental prostheses; PMMA: polymethylmethacrylate; PEEK: polyetheretherketone; PEKK: polyetherketoneketone.

### Tooth preparation

#### Pre-preparation putty index fabrication

Before tooth preparation, a sectional putty index that could be reconstructed over the original tooth was made using a VPS impression material (Perfit^®^ Putty, Huge Dent, Shandong, China). The base and catalyst pastes of the putty impression material were proportioned and mixed according to the manufacturer’s instructions, reaching a homogeneous mix. A single putty index was made for each resin block or provisional FDP to serve as a reference for evaluating the required amount and uniformity of tooth reduction for both abutment teeth [19, 20].

#### Occlusal preparation

The selected teeth were prepared occlusally through a freehand technique using a high-speed handpiece under constant copious water cooling. The preparation started by making occlusal guiding grooves using a standard-grit tapered diamond bur with a round end (TR-16, AZDENT^®^ Zhengzhou, China). The depth of these grooves was 1.3 mm for functional palatal cusps and 0.8 mm for non-functional buccal cusps. After marking the depth of the prepared grooves using a waterproof marker, the tapered diamond bur with a round end was used to remove the islands of tooth structure in between, following the occlusal anatomic configuration until the occlusal surface was uniformly reduced, leaving 0.2 mm for the finishing step.

#### Axial preparation

In this study, a dental surveyor (Milling unit BF 2^®^, Bredent GmbH & Co., Senden, Germany) was used to perform a standardized axial preparation for all selected natural teeth and maintain a consistent convergence angle and uniform path of insertion [21]. The finish line location of each tooth was defined with a waterproof marker 1 mm coronal to the CEJ level. This procedure included using the tapered diamond bur with a round end (TR-16, AZDENT^®^, Zhengzhou, China) to make a 1 mm axial reduction with 6°-tapered axial surfaces following the original contour and a circumferential 0.8 mm chamfer finish line.

#### Finishing the preparation

**Figure 1:**
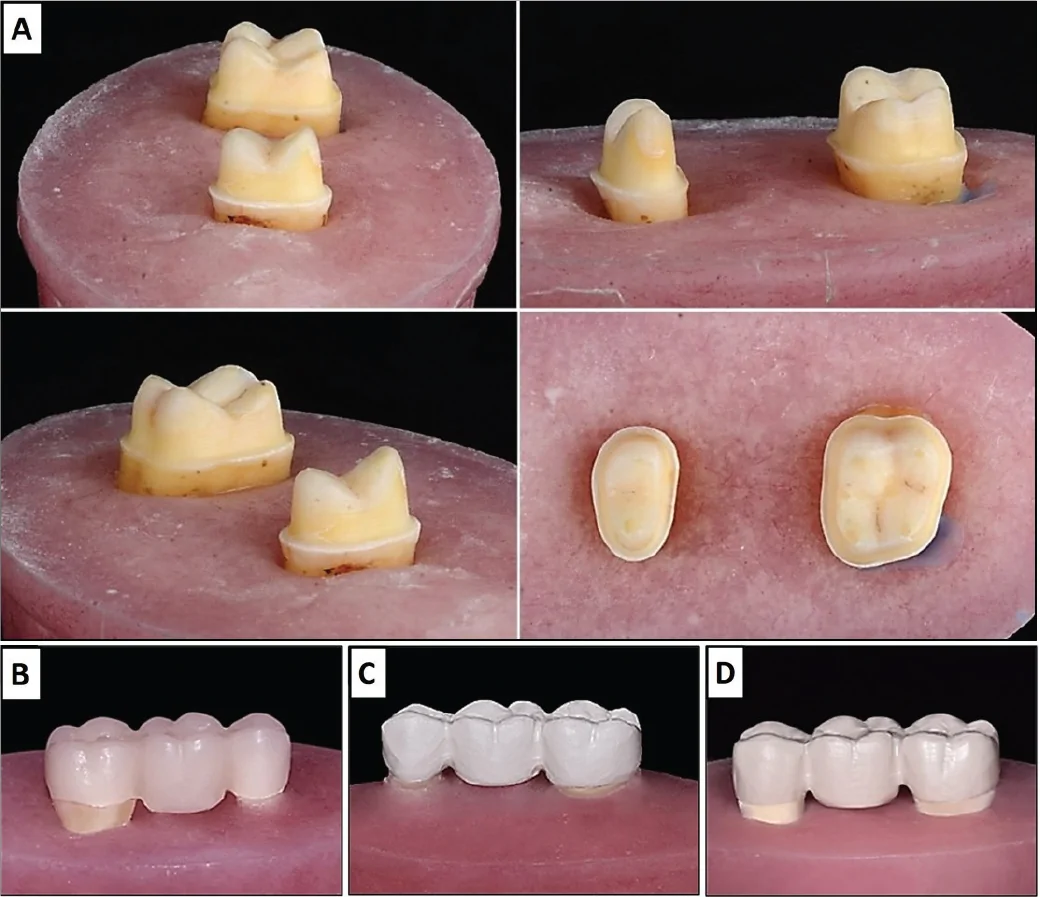
Standardized prepared abutment teeth following the finishing procedure (A) and cemented provisional FDPs for all studied groups: PMMA (B), PEEK (C), and PEKK (D).

All prepared surfaces were properly finished and smoothed using fine-grit diamond burs to eliminate any contour irregularity, bur striations or sharp line angles that might serve as focal points of stress concentration. The axial preparation was evaluated to ensure the removal of any undercut area and the maintenance of axial surfaces with smooth, rounded line angles and a smooth regular, even-thickness finish line. The finished prepared teeth were featured with 1.5 mm occlusal reduction for functional palatal cusps, 1 mm occlusal reduction for non-functional buccal cusps, 1 mm axial reduction, and 0.8 mm circumferential chamfer finish line (Figure 1A). The amount and uniformity of preparation were verified through the putty index fabricated before preparation. All preparations were performed by a single experienced operator under dental loupe magnification [19].

### Provisional FDPs fabrication

Based on specimen grouping, a total of 24 three-unit provisional FDPs were CAD/CAM-fabricated. A digital workflow consisting of scanning (computer-aided impression, CAI), designing (CAD), and milling (CAM) phases was followed.

#### Scanning phase (CAI)

After the teeth preparation was successfully finished, proper steam cleaning and complete drying were applied to all prepared teeth. Each numbered block was individually scanned using a highly sensitive 3D optical scanner (Medit T310^®^, Medit Corp., Seoul, Korea). High-resolution scan data were obtained using dedicated scanning software (colLab^TM^ Scan, Medit^®^ Corp., Seoul, Korea).

#### Designing phase (CAD)

Using CAD software (DentalDB 3.1 Rijeka, exocad^®^ GmbH, Darmstadt, Germany), a three-unit FDP was designed for each scanned acrylic resin block. Following the manufacturer’s recommendations, the CAD design parameters were standardized for all specimens, including a cement space of 40 µm, the FDP path of insertion, and the prosthesis morphology, until the final full-anatomical FDP design was obtained. Ridge lap pontics and connector dimensions of 12 mm² (3 mm width × 4 mm height) were incorporated into the design. To ensure morphological standardization, the completed design of the first three-unit FDP served as the master reference model and was subsequently applied to all specimens. Accordingly, the same design parameters, including the cement space, connector dimensions, occlusal morphology, and prosthesis thickness, were consistently maintained across all experimental groups.

#### Milling phase (CAM)

Following validation of the virtual FDP design, the finalized restoration file was nested using a dental CAM software (iCAM V5 Smart^TM^, imes-icore^®^ GmbH, Eiterfeld, Germany). After completion of tool-path generation, the dry milling process was performed using a five-axis milling unit (CORiTEC 150i PRO, imes-icore GmbH, Eiterfeld, Germany) equipped with manufacturer-recommended milling burs suitable for both gross milling and final refinement.

Upon completion of milling, each FDP was carefully detached from the blank using a diamond bur. Only minimal finishing was performed to smooth the attachment areas, followed by final finishing and high-gloss polishing using manufacturer-specific polishing systems under low rotational speed and light pressure. Sequential silicone/rubber polishers (coarse to fine grit) and final polishing paste with a soft goat-hair brush were used. The thickness of all provisional FDPs was verified after milling using a dental precision measuring caliper (Calipretto-S^®^ precision measuring instrument 1122-1000, Renfert GmbH, Hilzingen, Germany) at predefined reference points, including the occlusal surface, connector area, and axial walls, to ensure consistency in prosthesis dimensions across all specimens. Subsequently, each FDP was tried on its corresponding resin block, cleaned using gentle steam spray, and thoroughly dried.

### Pre-cementation marginal adaptation testing

The specimens of all studied groups were evaluated using a calibrated stereomicroscope (SZ61TR, Model SZ2-ILST, Olympus^®^ Co., Tokyo, Japan) at up to 40× magnification to evaluate the level of marginal adaptation or fit prior to the cementation procedure. A special digital image analysis software (ISCapture^®^ 4.1, Informer Technologies, Inc., California, USA) was used to determine the amount of marginal gap in microns (µm). Before each measurement session, the stereomicroscope was calibrated according to the manufacturer’s instructions. All measurements were performed by the same experienced examiner, who was extensively trained in operating the stereomicroscope and digital image analysis software. The examiner was blinded to the group allocation throughout the measurement procedures. A standardized measurement protocol was followed to ensure measurement consistency. Three standardized measurement points were selected on each surface of each retainer. At each measurement point, three consecutive readings were recorded, and their mean value was calculated to minimize random measurement error. Subsequently, the mean values of the three measurement points were averaged to obtain the marginal gap value for each surface, and the mean values of all evaluated surfaces were then averaged to determine the overall pre-cementation marginal gap value for each FDP [22, 23]. Based on previous reports, marginal discrepancies of less than 120 μm were considered clinically acceptable [6, 19].

### Provisional FDPs cementation

According to specimen grouping, each provisional FDP was cemented to its corresponding prepared abutment teeth (Figure 1B–D). Because of its widespread clinical use, ease of retrieval, and compatibility with both provisional and subsequent definitive restorations, a type of two-paste, zinc oxide eugenol-free temporary cement (Cavex^®^ Temporary Cement, Cavex Holland BV, Haarlem, Netherlands) was used according to the manufacturer’s instructions [24]. The same cement was used for all specimens to standardize the cementation procedure and control cement type as a potential confounding variable. To standardize seating force, the specimens were sequentially inserted into a custom-made loading device. An axial load of 5 kg was applied centrally and perpendicular to the occlusal surface [12]. The excess cement was removed when it felt rubbery (after 2 min) using a dental probe, followed by marginal finishing and polishing approximately 5 min after seating of the FDP restoration. Finally, all cemented specimens were stored and preserved in the saline solution for 24 h prior to testing [12, 25].

### Fatigue testing

#### Thermal cycling testing (thermal aging)

To reproduce the thermal stresses encountered in the oral environment, all specimens were subjected to accelerated artificial aging (AAA) using a thermocycler (SD Mechatronik GmbH, Feldkirchen-Westerham, Germany). This thermal aging consisted of 10,000 cycles between water baths maintained at 5 ± 1°C and 55 ± 1°C. Each cycle included a dwell time of 30 s in each bath and a transfer time of 5 s between baths. This protocol corresponds to approximately 1 year of clinical service and was conducted according to the recommendations of the International Organization for Standardization (ISO/TS 11405:2015) [12].

#### Dynamic loading testing (mechanical aging)

All cemented specimens underwent 240,000 loading cycles utilizing a chewing simulator device (CS-4.4^TM^, SD Mechatronik GmbH, Feldkirchen-Westerham, Germany), designed to simulate 1 year of oral function. This cyclic loading was applied at a frequency of 1.6 Hz with an axial load magnitude of 50 N. The specimens were loaded while in distilled water at 37°C using a steatite-ceramic (enamel-like), ball-shaped antagonist, 6 mm in diameter. The ball was positioned at the central point of the pontic’s occlusal surface, contacting the buccal and palatal cusps, while being aligned parallel to the long axes of the teeth. The integrity of specimens was periodically assessed through dental loupe inspection under adequate illumination to identify any early signs of failure [11, 14].

### Post-cementation marginal adaptation testing

Following the aging process, marginal adaptation was reevaluated for all specimens using the same calibrated stereomicroscope and digital image analysis software, following the identical standardized measurement protocol employed in the pre-cementation assessment. Measurements were obtained by the same experienced, blinded examiner at the same predefined measurement points on each surface of each retainer. Three consecutive readings were recorded at each measurement point, and their mean value was calculated. The mean values were subsequently averaged at the surface and restoration levels to ensure methodological consistency and comparability with the baseline measurements.

### Fracture resistance testing

All specimens underwent fracture resistance testing using a computer-controlled, dual-column, tabletop universal testing machine (Model 3365, Instron^®^, Norwood, MA, USA). Each sample was positioned on the lower fixed platform, while a metal loading stylus was attached to the upper movable part of the machine. A compressive load of 5 kN was axially and centrally applied using a stainless steel loading plate, ensuring uniform contact across the occlusal surfaces of the three-unit FDP and allowing even distribution of compressive forces during testing. Loading was applied at a crosshead speed of 1 mm/min until fracture or failure of the sample occurred. The maximum fracture load was recorded in newtons (N), with failure identified by an audible cracking sound and confirmed by a sudden drop in the load-deflection curve generated by Bluehill^®^ software (Instron^®^, Norwood, MA, USA) [25–27].

### Fractographic analysis

Based on three-examiner consensus, the fractured specimens were qualitatively evaluated to determine the resulting fracture types and categorize the failure modes for all tested groups as either adhesive (failure at FDP/abutment teeth interface), cohesive (failure within FDP or/and abutment teeth), or mixed (both types) [28]. For a detailed and highly defined analysis, representative specimens from each failure category for all studied groups were ultrasonically cleaned in isopropyl alcohol for 10 min, followed by distilled water for an additional 10 min, to remove all impurities. Subsequently, dried specimens were sputter-coated with a uniform 30 nm gold layer for 180 s at 40 mA using a Sputter Coating Evaporator (SPI Module^TM^ – Sputter Gold/Carbon Coater, SPI Supplies^®^, Structure Probe, Inc., West Chester, PA, USA) [29, 30]. The coated specimens were examined using Scanning Electron Microscopy (SEM) (JSM.6510LV, JEOL^®^ Ltd., Tokyo, Japan), and images of the fractured surfaces were captured and documented at various magnifications according to the region of interest. Finally, these SEM images were analyzed to identify the mode of failure based on systematic mapping of the fracture origin(s) and the overall direction of crack propagation for all studied groups [11, 14, 31–33].

### Statistical analysis

The data obtained were analyzed using SPSS^®^ (Statistical Package for Social Sciences) statistical software (version 22, IBM^®^ Co., Armonk, NY, USA). Quantitative data were presented as mean values and standard deviations (SD) after verification of normal distribution using the Kolmogorov–Smirnov test. Marginal adaptation data were analyzed using a two-way repeated-measures analysis of variance (ANOVA) (Material type × Testing time), with material type as the between-subject factor and testing time (before cementation, after cementation, and aging) as the within-subject factor. Fracture resistance data were analyzed using one-way ANOVA. Effect sizes (partial eta-squared, η²p) were calculated for both ANOVA models to estimate the magnitude of the observed effects. When significant main effects were detected, Tukey’s post hoc test was used for pairwise comparisons. Qualitative data were expressed as numbers and percentages with intergroup comparisons using Fisher’s exact test. Statistical significance of the reported results was set at *P* ≤ 0.05.

## Results

### Marginal adaptation

A two-way repeated-measures ANOVA was performed to evaluate the effects of material type, testing time, and their interaction on marginal adaptation (Table 2). The analysis demonstrated a highly significant main effect of material type (*P* < 0.001, η²p = 0.524), indicating significant differences in marginal adaptation among the tested restorative materials. A significant main effect of testing time was also observed (*P* = 0.003, η²p = 0.348), indicating that marginal adaptation differed significantly between the two measurement time points. Furthermore, a significant interaction between material type and testing time was identified (*P* = 0.021, η²p = 0.233), indicating that the effect of testing time on marginal adaptation depended on and differed according to the restorative material.

**Table 2:** Two-way repeated-measures ANOVA results for marginal adaptation (µm).

Parameter	Source	Type III Sum of Squares	df	Mean Square	*F*	*P*	Partial eta-squared (η²p)^†^
Marginal adaptation	Material type	55.45	2	27.73	11.58	< 0.001*	0.524 (Large)
	Testing time	18.26	1	18.26	11.23	0.003*	0.348 (Large)
	Material type × Testing time	10.35	2	5.18	3.19	0.021*	0.233 (Large)

(*) Significant at *P*-value ≤ 0.05;

(†) Partial eta-squared values of 0.01, 0.06, and 0.14 indicate small, medium, and large effect sizes, respectively.

Mean marginal adaptation values (µm) before cementation and after cementation and aging for all studied groups, together with Tukey’s post hoc comparisons, are presented in Table 3. Before cementation, Tukey’s post hoc analysis identified a statistically significant difference between PMMA and PEKK, with PEKK demonstrating greater marginal discrepancy. No statistically significant differences were detected between PMMA and PEEK or between PEEK and PEKK. After cementation and aging, Tukey’s post hoc comparisons revealed that PMMA exhibited significantly lower marginal discrepancy compared with both PEEK and PEKK, while no statistically significant difference was observed between PEEK and PEKK.

**Table 3:** Descriptive statistics (Mean ± SD) of marginal adaptation (µm) with Tukey’s post hoc comparisons among studied groups.

Group	*n*	Testing time	Mean ± SD
PMMA	8	Before cementation	38.5 ± 3.1^**A**^
		After cementation and aging	36.8 ± 1.2^**a**^
PEEK	8	Before cementation	40.7 ± 1.9^**AB**^
		After cementation and aging	38.9 ± 2.1^**b**^
PEKK	8	Before cementation	41.0 ± 1.3^**B**^
		After cementation and aging	40.1 ± 1.2^**b**^

(AB, ab) Tukey’s post hoc test: values with the same superscripted letters represent non-significant differences, while different superscripted letters represent significant one (**AB**: significance before cementation, **ab**: significance after cementation and aging).

SD: Standard Deviation; PMMA: polymethylmethacrylate; PEEK: polyetheretherketone; PEKK: polyetherketoneketone.

### Fracture resistance

The highest mean fracture resistance value (± SD) was recorded for PEKK (2,669 ± 204 N), followed by PEEK (1,849 ± 183 N), whereas PMMA demonstrated the lowest mean value (1,072 ± 127 N). One-way ANOVA revealed a statistically significant difference in fracture resistance among the tested groups (*P* < 0.001, η²p = 0.941), indicating a significant and large effect of material type on fracture resistance (Table 4).

**Table 4:** Descriptive statistics (Mean ± SD) with one-way ANOVA test and Tukey’s post hoc test for comparison of fracture resistance (N) among studied groups.

Group	*n*	Mean ± SD	One-way ANOVA	Partial eta-squared (η²p)^†^
PMMA	8	1,072 ± 127^**a**^	*F* = 168.33 *P* < 0.001*	0.941 (Large)
PEEK	8	1,849 ± 183^**b**^		
PEKK	8	2,669 ± 204^**c**^		

(abc) Tukey’s post hoc test: values with different superscripted letters represent significant differences;

(*) Significant at *P*-value ≤ 0.05;

(†) Partial eta-squared values of 0.01, 0.06, and 0.14 indicate small, medium, and large effect sizes, respectively.

SD: Standard Deviation; PMMA: polymethylmethacrylate; PEEK: polyetheretherketone; PEKK: polyetherketoneketone.

Multiple comparisons using Tukey’s post hoc test demonstrated statistically significant differences among all pairwise comparisons. PEKK showed significantly higher fracture resistance than both PEEK and PMMA (*P* < 0.001). Additionally, PEEK exhibited significantly higher fracture resistance than PMMA (*P* < 0.001). These findings indicate a clear gradation in fracture resistance among the investigated materials (Table 4).

The distribution of fracture modes among tested groups is presented in Table 5. Fisher’s exact test revealed a statistically significant difference between the studied groups (*P* = 0.019). PMMA and PEEK groups predominantly exhibited mixed failure modes, whereas PEKK showed mainly adhesive failures. Cohesive failure was rare and observed only in the PMMA group. Overall, mixed failure was the most common mode, followed by adhesive and cohesive failures.

**Table 5:** Distribution of failure modes among studied groups with intergroup comparison using Fisher’s exact test.

Group	*n*	Failure mode			Fisher’s exact test
		Adhesive (%)	Cohesive (%)	Mixed (%)	
PMMA	8	0 (0%)	1 (12.5%)	7 (87.5%)	*P* = 0.019*
PEEK	8	2 (25%)	0 (0%)	6 (75%)	
PEKK	8	6 (75%)	0 (0%)	2 (25%)	
Total	24	8 (33.3%)	1 (4.2%)	15 (62.5%)	

(*) Significant at *P*-value ≤ 0.05.

PMMA: polymethylmethacrylate; PEEK: polyetheretherketone; PEKK: polyetherketoneketone.

**Figure 2:**
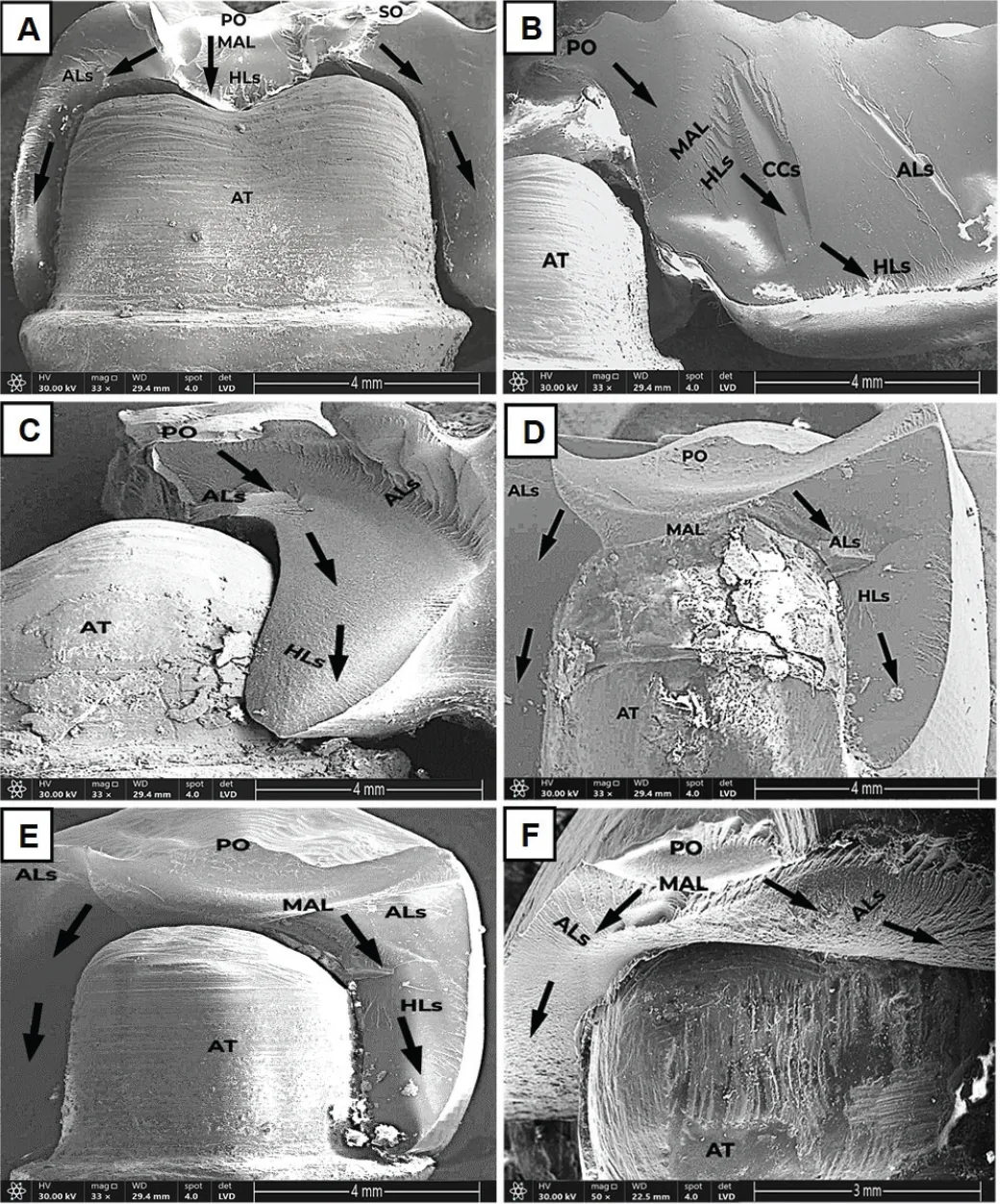
Representative SEM images (33× and 50×) for fractographic analysis of all tested groups: (A and B) PMMA, (C and D) PEEK, and (E and F) PEKK. PO: primary origin; SO: secondary origin; MAL: main arrest line; ALs: arrest lines; HLs: hackle lines; CCs: compression curls; AT: abutment tooth; black arrows: direction of crack propagation.

SEM fractographic analysis of all tested groups (Figure 2) demonstrated that the primary fracture origin (PO) was consistently located on the occlusal surface of the retainer(s) and/or the pontic, directly beneath the main occlusal loading area applied during the fracture resistance test. Crack propagation progressed in a corono-apical direction, as evidenced by the presence of hackle lines (HLs) and main arrest lines (MALs) that clearly indicated the direction and path of crack advancement. In some specimens, additional minor or secondary fracture origins (SO) were observed on the occlusal surface. Tooth fractures were also detected in some specimens across all groups. Furthermore, diffuse and well-defined arrest lines (ALs), accompanied by marked surface irregularities, were identified on the fractured surfaces, especially for PEEK and PEKK groups. These fractographic features are typical of polymer-based materials, reflecting the accumulation of high elastic energy prior to fracture. Compression curls (CCs), indicative of crack deflection and re-initiation before final failure, were likewise observed.

## Discussion

Despite promising properties, the available literature directly comparing PEKK with PEEK and PMMA in terms of marginal adaptation and fracture resistance remains limited. The present in vitro study was designed to evaluate and compare the marginal adaptation and fracture resistance of CAD/CAM-fabricated three-unit provisional FDPs constructed from PMMA, PEEK, and PEKK materials. The null hypothesis was rejected, as statistically significant differences were detected among the tested materials regarding both marginal adaptation and fracture resistance. Overall, PMMA material exhibited the most favorable marginal adaptation but the lowest fracture resistance. In contrast, PEKK material demonstrated the highest fracture resistance, despite showing relatively greater marginal discrepancies. PEEK material provided a balanced performance in terms of marginal adaptation and fracture resistance.

The choice of materials (PMMA, PEEK, and PEKK) was deliberate. PMMA was included as a conventional reference material due to its widespread use in provisional restorations, despite its known limitations such as polymerization shrinkage, brittleness, and poor color stability [32]. PEEK and PEKK, on the other hand, were selected as representatives of high-performance polymers. Their superior mechanical properties, biocompatibility, and adaptability to CAD/CAM fabrication make them promising alternatives for long-term provisional and definitive restorations. CAD/CAM milling from industrially pre-polymerized blocks minimized porosity and improved homogeneity compared to chairside-mixed resins, ensuring that observed differences were primarily attributable to intrinsic material properties rather than fabrication inconsistencies [34–36].

The testing methods selected for evaluating marginal adaptation and fracture resistance are well-established and validated in the dental literature. Marginal discrepancy assessment provides insight into the sealing ability of provisional restorations, which is crucial for preventing microleakage and maintaining pulpal health [37, 38]. Similarly, fracture resistance testing offers valuable information regarding the ability of materials to withstand functional and parafunctional loads [39–41]. The combination of these outcome measures provides a comprehensive evaluation of the clinical performance of PMMA, PEEK, and PEKK provisional restorations.

Marginal adaptation is a critical determinant for the long-term success of FDPs, as increased marginal discrepancy has been associated with cement dissolution, microleakage, secondary caries, and periodontal complications [2]. In the current study, marginal adaptation values for all tested materials were within the clinically acceptable range (< 120 μm), which is consistent with previous investigations on CAD/CAM polymer-based restorations [1, 10, 19]. Moreover, the effects of material type, testing time, and their interaction were associated with large effect sizes, indicating that these factors had not only statistically significant but also clinically meaningful influences on marginal adaptation.

Before cementation, PMMA demonstrated the lowest marginal discrepancy, followed by PEEK, while PEKK exhibited the highest values. A statistically significant difference was detected between PMMA and PEKK, whereas no significant differences were observed between PMMA and PEEK or between PEEK and PEKK. The observed baseline differences may reflect the combined influence of intrinsic material characteristics and CAD/CAM processing. From a processing perspective, variations in milling behavior during the CAD/CAM manufacturing procedure may have contributed to the observed dimensional differences among the tested materials. From a material perspective, PMMA possesses a relatively homogeneous amorphous structure and lower stiffness, which have been reported to facilitate smoother and more accurate milling behavior compared with high-performance polymer blocks [19].

After cementation, PMMA continued to exhibit significantly superior marginal adaptation compared to both PEEK and PEKK. Interestingly, PEEK was the only material that showed a statistically significant improvement in marginal adaptation after cementation. According to previous literature and standard polymer profiles, this behavior is likely associated with the specific viscoelastic and compliant nature reported for PEEK, which may facilitate more favorable sealing and adaptation dynamics under the standardized cementation and aging conditions used in the present study [42]. Similar findings were reported by Zoidis et al. [43], who observed improved marginal fit of PEEK restorations following cementation due to their resilient nature.

In contrast, PEKK did not demonstrate a statistically significant improvement after cementation. This finding might be related to the higher elastic modulus and increased rigidity reported for PEKK compared with PEEK in standard material profiles, which could potentially limit its ability to adapt under the standardized cementation conditions used in this study [44]. These results are in agreement with the findings of Stawarczyk et al. [8], who reported higher marginal discrepancies for PEKK frameworks compared to PEEK. However, other studies have reported contradictory results, suggesting comparable marginal adaptation between PEEK and PEKK restorations. Such discrepancies may be explained by variations in study design, cement type, measurement techniques, restoration span, or CAD/CAM systems used [23].

Fracture resistance is another essential factor influencing the clinical performance of FDPs, particularly in posterior regions subjected to high occlusal forces [39]. In the present study, PEKK exhibited the highest fracture resistance, followed by PEEK, while PMMA showed the lowest values. Highly statistically significant differences among all tested groups were revealed. These differences were also associated with a large effect size, confirming that the restorative material had a substantial influence on fracture resistance beyond statistical significance.

Although the intrinsic mechanical properties of the tested materials were not directly evaluated in the present study, the superior fracture resistance of PEKK and PEEK is consistent with their mechanical behavior previously reported in the literature. The higher fracture load recorded for PEKK compared with PEEK may be cautiously attributed to its reported chemical architecture, which incorporates a higher ketone-to-ether ratio that potentially provides a more rigid polymeric backbone and higher resistance to crack propagation than PEEK [34]. These findings are consistent with previous studies that demonstrated significantly higher fracture resistance of PEKK restorations compared to PEEK and PMMA [8, 45].

PEEK also demonstrated significantly higher fracture resistance than PMMA, which aligns with previous investigations highlighting the favorable mechanical performance of PEEK in fixed prosthodontics [39]. In contrast, PMMA showed the lowest fracture resistance values, which could be related to its intrinsically lower molecular weight and lack of reinforcing polymeric networks, as documented in standard material profiles, making it more prone to brittle fracture under simulated masticatory forces [46].

Although PMMA exhibited inferior fracture resistance, its values still exceeded the average masticatory forces reported in the posterior region, which range between 500 and 900 N [47]. These findings indicate that all tested materials withstood static loads exceeding the average posterior masticatory forces reported in the literature under the present experimental conditions. PEKK and PEEK demonstrated significantly higher fracture resistance than PMMA, suggesting greater mechanical potential for provisional FDP applications. However, fracture resistance under monotonic loading should not be considered the sole predictor of long-term clinical performance, which is also influenced by fatigue behavior, wear, hydrolytic degradation, and repeated functional loading. Some studies, however, reported no significant difference in fracture resistance between PEEK and PEKK frameworks. These conflicting findings may reflect methodological differences among studies, including variations in connector dimensions, veneering materials, aging protocols, and loading conditions [42].

The present study revealed a statistically significant difference in fracture mode distribution among the tested CAD/CAM provisional FDP materials. PMMA restorations predominantly exhibited mixed failure (87.5%), with limited cohesive failure and absence of adhesive failure. This behavior is consistent with the relatively low fracture toughness and brittle nature typically associated with PMMA, which potentially increases susceptibility to crack initiation and propagation within the restoration as well as at the cement–restoration interface, particularly in multi-unit FDPs [37, 45].

In contrast, the PEEK group demonstrated a combination of mixed (75%) and adhesive (25%) failure modes, with no cohesive failures observed. The lack of cohesive fracture indicates adequate intrinsic strength of the material. However, the low elastic modulus documented for PEEK, which is closer to that of dentin, is believed to allow elastic deformation under occlusal loading, potentially leading to stress concentration at the cement interface and subsequent adhesive failure. This stress distribution pattern has been reported to reduce catastrophic material fracture while promoting more favorable failure modes [34, 44].

PEKK restorations showed a distinct fracture behavior, with adhesive failure being the predominant mode (75%), followed by mixed failure (25%), and no cohesive failures. This finding could be explained by the superior mechanical properties reported for PEKK, including higher fracture resistance, increased toughness, and improved fatigue performance compared with PMMA and PEEK [34, 47]. Consequently, stresses may have been preferentially transferred to the luting cement rather than inducing internal fracture within the restoration, thereby preserving the structural integrity of the FDP [35].

Clinically, the predominance of adhesive failure observed in PEKK and PEEK groups may be advantageous, as adhesive failures are generally less destructive and may allow for re-cementation without compromising the restoration or abutment teeth. In contrast, mixed or cohesive failures, as observed mainly in PMMA restorations, are more likely to necessitate replacement of the provisional prosthesis [28]. Therefore, the fracture mode findings of the present study further support the potential clinical benefits of using high-performance polymers, particularly PEKK, for CAD/CAM-fabricated multi-unit provisional FDPs subjected to functional loading.

Several limitations of the present study must be acknowledged. Although all specimens were subjected to standardized thermomechanical aging, including thermal cycling and cyclic mechanical loading, prior to fracture testing, the primary mechanical outcome of this study was the ultimate fracture load obtained under monotonic loading conditions. Therefore, the fracture resistance findings should be interpreted with caution, as they do not directly reflect the fatigue behavior or long-term clinical performance of provisional FDPs under continuous cyclic functional loading. Furthermore, the study was conducted under in vitro conditions, which do not fully replicate the complex oral environment, including variations in saliva composition, thermal fluctuations, occlusal wear, hydrolytic degradation, and long-term functional loading. In addition, the use of a single CAD/CAM system, one cement type, a single connector design, one prosthesis geometry, and the focus on three-unit FDPs may limit the generalizability of the present findings. The relatively small sample size may also limit the statistical power and external validity of the study. Accordingly, the present findings should be interpreted as preliminary laboratory evidence rather than definitive guidance for clinical material selection.

Future research should address these limitations by incorporating larger sample sizes, diverse CAD/CAM systems, and extended cyclic fatigue testing protocols to better simulate long-term clinical function. Long-term clinical trials are also necessary to evaluate the performance of PMMA, PEEK, and PEKK restorations under actual oral conditions. Comparative studies involving different cement types, connector designs, prosthesis geometries, restorative materials, and aging protocols would provide a more comprehensive understanding of material-specific behavior. Additionally, exploring advanced fabrication techniques, such as hybrid CAD/CAM–3D printing approaches, may open new possibilities for optimizing the performance of high-performance polymer restorations.

The present findings have relevant clinical implications for prosthodontic practice. CAD/CAM PMMA restorations demonstrated superior marginal adaptation, which may help reduce plaque accumulation, the risk of secondary caries, and periodontal complications, thereby enhancing patient comfort and long-term tooth preservation. In contrast, PEKK and PEEK restorations exhibited higher fracture resistance, suggesting their potential use in patients with high occlusal loads, parafunctional habits, or prolonged provisionalization. These findings may assist clinicians in selecting provisional restorative materials according to the clinical context, functional requirements, and anticipated longevity of the prosthesis. CAD/CAM PMMA may offer advantages in terms of marginal adaptation, whereas PEKK and PEEK may be considered when mechanical strength is the primary requirement.

## Conclusions

Within the limitations of this in vitro study and under the tested experimental conditions, the type of CAD/CAM material significantly affected both the marginal adaptation and fracture resistance of three-unit provisional FDPs. All tested PMMA, PEEK, and PEKK materials demonstrated marginal adaptation within the clinically acceptable range and fracture resistance values exceeding the normal posterior masticatory forces. Among the tested materials, PMMA material demonstrated the lowest marginal discrepancy but the lowest fracture resistance. In contrast, PEKK material exhibited the highest fracture resistance but relatively greater marginal discrepancies. PEEK material provided a balanced performance in terms of marginal adaptation and fracture resistance.

## Data Availability

The datasets generated and/or analyzed dring the current study are available from the corresponding author upon reasonable request.

## Permission to reproduce material from other sources

No material from other sources was reproduced in this manuscript.
